# Opioid-free anesthesia in enhanced recovery after surgery for gastrointestinal surgery: current status, challenges, and prospects

**DOI:** 10.3389/fphar.2025.1662818

**Published:** 2025-09-10

**Authors:** Yongxing Xu, Maolin Zhong, Shihong Li

**Affiliations:** ^1^ The First Clinical Medical College of Gannan Medical University, Ganzhou, China; ^2^ Department of Anesthesiology, First Affiliated Hospital of Gannan Medical University, Ganzhou, China; ^3^ Ganzhou Key Laboratory of Anesthesiology, Ganzhou, China

**Keywords:** opioids, opioid-free anesthesia, enhanced recovery after gastrointestinal surgery, laparoscopic surgical, abdominal surgery analgesia

## Abstract

The enhanced recovery after surgery (ERAS) protocol has been increasing implementation in gastrointestinal surgeries to optimize perioperative management, mitigate surgical stress responses, and accelerate patient recovery. Although opioid-based anesthesia effectively alleviates pain, it is associated with significant adverse effects, including postoperative nausea and vomiting (PONV), respiratory depression, and intestinal paralysis, which can impeder early recovery. Opioid-free anesthesia (OFA) is designed to alleviate these concerns. This article examines the pharmacological agents and regional block techniques commonly employed in OFA, emphasizing its role in promoting the recovery of gastrointestinal function, improving pain management, reducing adverse events, and enhancing patient satisfaction.

## 1 Introduction

With the continuous advancement of modern medicine, enhanced recovery after surgery (ERAS) has emerged as an innovative surgical concept and has become a focal point of research and practice in gastrointestinal surgery. ERAS aims to optimize various perioperative management strategies, encompassing preoperative patient education and nutritional support, precision anesthesia, meticulous surgical techniques, early postoperative mobilization, and effective pain management. Collectively, these measures mitigate surgical trauma and stress responses, thereby expediting recovery, reducing postoperative complications and mortality, shortening hospital stays, lowering medical costs, and alleviating the social and familial burden associated with surgery (Ni et al., 2019; Scott et al., 2015; Feldheiser et al., 2016).

Traditionally, opioids have played a central role in anesthesia and pain management during the perioperative period of gastrointestinal surgeries. These agents provide potent analgesia, effectively alleviating surgical pain and facilitating smooth procedural conduct. However, with their expanded clinical use, the limitations of opioids have become increasingly evident. Adverse effects such as respiratory depression, which can lead to hypoxemia and compromise respiratory function and patient safety, are of significant concern. Postoperative nausea and vomiting (PONV) are also common, diminishing patient comfort and hindering oral intake and nutritional absorption. Constipation, another frequent side effect, often leads to abdominal distension and discomfort, prolonging recovery time. Moreover, long-term opioid use poses risks of addiction, which can profoundly affect patients’physical and mental health (de Boer et al., 2017; Paul et al., 2021). These adverse effects not only impede the quality of postoperative recovery but also increase medical risks and patient suffering, highlighting the critical need for safer and more effective anesthetic approaches in gastrointestinal ERAS.

In response to these challenges, opioid-free anesthesia (OFA) has been developed. OFA minimizes or eliminates opioid use by employing a combination of non-opioid pharmacologic agents and techniques to achieve effective anesthesia and analgesia while reducing the incidence of opioid-related adverse effects. Increasingly recognized in clinical practice, OFA is now widely implemented across various surgical procedures. It has demonstrated efficacy in managing perioperative pain, reducing opioid consumption, accelerating postoperative recovery, and shortening hospital stays (Feng et al., 2024; Liu et al., 2023; Zhou et al., 2023; Cha et al., 2023; Hao et al., 2023; Zhang Q. et al., 2023; Wang et al., 2024).

The introduction of OFA marks a significant advancement in gastrointestinal ERAS, addressing many limitations associated with opioid use while better aligning with patient needs. This approach not only enhances recovery outcomes but also fosters further progress in the field. This review aims to provide a comprehensive overview of the clinical applications of OFA in gastrointestinal ERAS, offering evidence-based insights to guide clinical practice.

## 2 OFA: an overview of commonly used drugs and regional block strategies

### 2.1 Commonly used drugs

Drugs used in OFA are primarily categorized into four groups based on their mechanisms of action: α2-adrenergic agonists (e.g., clonidine and dexmedetomidine); Sodium channel blockers (e.g., lidocaine); NMDA receptor antagonists (e.g., esketamine); Nonsteroidal anti-inflammatory drugs (NSAIDs). Each category contributes distinct pharmacological properties to OFA. We provide an overview of these agents below.

#### 2.1.1 Dexmedetomidine

Dexmedetomidine is a highly selective α2-adrenergic receptor agonist with sedative, anxiolytic, analgesic, sympatholytic, and opioid-sparing properties (Hall et al., 2000; Scheinin et al., 1992). It induces a unique sedative state, enabling patients to transition smoothly between sleep and wakefulness. Notably, sedated patients remain responsive to stimuli and capable of communication, with minimal impact on respiratory function (Goettel et al., 2016).

In perioperative pain management, dexmedetomidine is widely employed across various surgical procedures to reduce opioid consumption, alleviate postoperative pain, and minimize adverse effects, thereby facilitating postoperative recovery (Zhong et al., 2024; Zheng et al., 2024; Zeng et al., 2025; Coeckelenbergh et al., 2021; Hu et al., 2021). For instance, Lu et al. reported that intraoperative administration of dexmedetomidine significantly improved postoperative gastrointestinal function recovery in elderly patients (Lu et al., 2021).

Beyond intravenous administration, dexmedetomidine is also used as an adjuvant to local anesthetics, extending their duration of action and further decreasing opioid requirements (Zhao et al., 2023; Yang et al., 2022). Marhofer et al. demonstrated that, during ultrasound-guided ulnar nerve blocks, dexmedetomidine as an adjuvant to local anesthetics resulted in faster onset and prolonged duration of nerve blockade compared to intravenous administration (Marhofer et al., 2013).

Due to its broad utility, dexmedetomidine is frequently combined with other non-opioid drugs in OFA protocols, yielding significant clinical benefits (Wang et al., 2024; Zhou et al., 2023; Berlier et al., 2022). However, a randomized controlled trial found that intraoperative use of dexmedetomidine in medium-to-large non-cardiac surgeries, while effective at reducing opioid consumption and postoperative nausea and vomiting, was associated with adverse events such as hypoxemia and bradycardia (Beloeil et al., 2021). These risks, including bradycardia and hypotension, can be mitigated with appropriate interventions, such as atropine or vasoactive agents (Ahn et al., 2016; Park et al., 2020).

To ensure safety, thorough preoperative evaluation and careful dosage adjustments are essential to prevent severe adverse events. In conclusion, dexmedetomidine, when used appropriately, serves as a valuable adjuvant in OFA, reducing adverse effects and enhancing postoperative recovery.

#### 2.1.2 Lidocaine

Lidocaine is an amide-type local anesthetic and antiarrhythmic drug that, beyond its traditional local anesthetic effects, also exhibits significant analgesic, anti-inflammatory, anti-hyperalgesic, and gastrointestinal motility-enhancing properties when administered intravenously (Beaussier et al., 2018; Hermanns et al., 2019). In clinical practice, intravenous lidocaine is used to reduce postoperative pain and opioid consumption, thereby decreasing the incidence of opioid-related adverse effects, including postoperative nausea, vomiting, and constipation (Kaba et al., 2007).

In OFA, lidocaine is frequently combined with other non-opioid drugs, such as dexmedetomidine and esketamine, to achieve enhanced analgesia and minimize postoperative complications. This combination has been shown to yield significant clinical benefits in various surgeries, where it significantly improves the overall recovery process for patients (Yu et al., 2023; Wang et al., 2023; Jose et al., 2023).

Despite its advantages, the use of lidocaine requires careful attention to safety. Excessive plasma concentrations can lead to severe toxic reactions, including suppression of the central nervous system (CNS) and cardiovascular system. Consequently, close monitoring of patients’ hemodynamic and neurological responses is critical to ensure both safety and efficacy. Additionally, for special populations such as elderly patients or those with renal impairment, individualized dose adjustments are essential to minimize the risk of adverse effects.

In summary, with appropriate dosing and monitoring, lidocaine serves as an effective adjuvant in OFA protocols, offering improved analgesia and a reduction in opioid-related complications, thereby enhancing postoperative recovery outcomes.

#### 2.1.3 Esketamine

Esketamine is a non-selective, non-competitive N-methyl-D-aspartate (NMDA) receptor antagonist with distinct properties, including analgesic, anesthetic, and antidepressant effects (Mion and Himmelseher, 2024). Clinically, esketamine is particularly effective for the induction of general anesthesia in short and minor surgical procedures, offering excellent analgesia while maintaining cardiovascular stability and enabling rapid emergence from anesthesia (Song et al., 2023).

In perioperative pain management, esketamine can be administered via various routes, including intravenous and epidural injection, to alleviate postoperative pain and reduce opioid consumption (Huan et al., 2025; Zhang Y. et al., 2023; Yan et al., 2023). Within OFA protocols, esketamine is often combined with other non-opioid agents, such as dexmedetomidine and lidocaine, to achieve multimodal anesthesia. This combination enhances postoperative pain control and reduces the incidence of opioid-related adverse effects, such as nausea, vomiting, and respiratory depression (Luo et al., 2025; Feng et al., 2024; Hao et al., 2023). Moreover, esketamine has been shown to promote postoperative gastrointestinal function recovery, shorten hospital stays, and improve the overall quality of recovery (Xu et al., 2023; Sun et al., 2023).

Despite its numerous benefits, careful management of esketamine is essential due to its potential adverse effects. These include neuropsychiatric symptoms, such as nightmares and hallucinations, as well as nausea, vomiting, and respiratory depression. To ensure safe and effective use, clinicians must tailor dosages to the patient’s specific condition and closely monitor their responses during administration.

In summary, esketamine serves as a novel and valuable anesthetic adjuvant in perioperative pain management. Its rational application within OFA protocols enhances postoperative recovery, reduces opioid reliance, and improves surgical outcomes.

#### 2.1.4 Non-steroidal anti-inflammatory drugs (NSAIDs)

NSAIDs are a class of medications that exert anti-inflammatory, analgesic, and antipyretic effects by inhibiting cyclooxygenase (COX) activity, thereby reducing prostaglandin synthesis. NSAIDs play a critical role in perioperative pain management as part of multimodal analgesia strategies, significantly reducing opioid consumption and related adverse effects.

Research has shown that flurbiprofen, a widely used NSAID, provides effective analgesia and opioid-sparing benefits across various surgical procedures. For instance, in esophagectomy, preoperative administration of flurbiprofen not only alleviates postoperative pain but also reduces opioid requirements, improves the oxygenation index, and lowers plasma IL-8 levels, thereby enhancing respiratory function and mitigating postoperative inflammatory responses (Wang et al., 2012). Similarly, in thyroid surgeries, a multimodal analgesic approach combining ropivacaine wound infiltration with flurbiprofen significantly reduces postoperative pain scores, decreases intraoperative remifentanil use, and avoids a rise in serious adverse events compared to tramadol alone (Li et al., 2019). Furthermore, in spinal fusion surgeries, preoperative flurbiprofen administration has been shown to significantly lower postoperative pain scores and morphine consumption, providing superior postoperative pain control (Yamashita et al., 2006).

Despite their demonstrated benefits, the use of NSAIDs in perioperative settings requires careful consideration. These drugs may increase the risk of gastrointestinal bleeding and renal complications and should be avoided in patients with a history of such conditions. Moreover, optimizing the timing and dosage of NSAID administration remains an area of ongoing research to maximize their efficacy and safety.

In conclusion, NSAIDs are a vital component of perioperative pain management. Their anti-inflammatory and analgesic properties effectively reduce opioid consumption and improve the quality of postoperative recovery. Future research should focus on further exploring their application in various surgical procedures and patient populations, as well as investigating their combination with other analgesics to achieve optimal pain relief with minimal adverse effects.

### 2.2 Regional block techniques

The introduction of the ERAS concept has placed increasing emphasis on early recovery, making the proactive adoption of multimodal analgesia strategies essential. Regional anesthesia, a cornerstone of multimodal analgesia, plays a pivotal role in achieving the objectives of ERAS. When combined with non-opioid analgesics, neuraxial anesthesia and peripheral nerve blocks effectively alleviate intraoperative and postoperative pain, facilitating low-opioid or OFA during the perioperative period.

Epidural anesthesia provides effective pain relief during and after thoracic, abdominal, and orthopedic surgeries. However, its use has declined due to the risk of severe complications, including catheter breakage, accidental subarachnoid injection, infection, and epidural hematoma (Rawal, 2021). With the popularization of ultrasound in clinical anesthesia practice, nerve block techniques have gradually been widely applied, and their safety has been ensured with the assistance of visualization technology. Peripheral nerve blocks involve the targeted delivery of local anesthetic solutions near specific nerves or nerve plexuses, achieving analgesia by reaching nerve fibers. Numerous studies have demonstrated that peripheral nerve blocks reduce perioperative opioid consumption and improve patient outcomes (Park et al., 2023; Dam et al., 2019; Lee et al., 2023; Pei et al., 2015).

Commonly used peripheral nerve blocks include.

• Upper limb surgeries: brachial plexus block.
• Thoracic and breast surgeries: pectoral nerve block, erector spinae plane block, and paravertebral block (Pei et al., 2015; Neethu et al., 2018; Zhang Q. et al., 2023).
• Abdominal surgeries: transversus abdominis plane block, quadratus lumborum block, erector spinae plane block, and lumbar plexus block (Park et al., 2023; Dam et al., 2019; Lee et al., 2013; Oksar et al., 2016).
• Lower limb surgeries: sciatic nerve block, fascia iliaca block, femoral nerve block, and adductor canal block (Luo et al., 2025).

Meanwhile, the addition of pharmacological adjuvants, such as dexmedetomidine or dexamethasone, to single-injection peripheral nerve blocks extends the duration of analgesia and further reduces opioid consumption (Marhofer et al., 2013; Zeng et al., 2024; Yang et al., 2022). Integrating regional block techniques within OFA protocols enhances analgesic efficacy and contributes significantly to improved patient outcomes, aligning with the principles of ERAS.

## 3 Clinical effects of OFA in gastrointestinal ERAS

### 3.1 Characteristics of gastrointestinal surgery

Gastrointestinal surgery (e.g., gastrectomy for gastric cancer, radical surgery for colorectal cancer) is defined by extensive trauma and significant disturbance to abdominal organs. The perioperative pathophysiological changes associated with these procedures primarily influence postoperative recovery in three critical aspects.

#### 3.1.1 Complexity of perioperative pain management

Gastrointestinal surgery involves abdominal wall incisions, manipulation of abdominal organs, and anastomotic procedures. Postoperative pain encompasses somatic pain (resulting from abdominal wall incisions), inflammatory pain, and visceral pain (due to gastrointestinal traction and bloating). The severity of pain correlates directly with the extent of surgical trauma, with laparoscopic surgery resulting in less pain compared to open surgery, although visceral pain remains a significant concern. While opioids effectively alleviate pain, their use is associated with adverse effects such as postoperative nausea and vomiting, constipation, and abdominal distension, all of which hinder recovery (de Boer et al., 2017; Paul et al., 2021).

Furthermore, advanced age and malnutrition, commonly observed in gastrointestinal surgery patients, increase the risk of opioid-induced respiratory depression. OFA addresses these challenges by utilizing multimodal analgesia, incorporating regional blocks (e.g., transversus abdominis plane block, quadratus lumborum block), NSAIDs, α2 receptor agonists (e.g., dexmedetomidine), and NMDA receptor antagonists (e.g., ketamine).

#### 3.1.2 Systemic inflammatory response induced by surgical trauma

Surgical trauma activates toll-like receptors and stimulates the release of inflammatory cytokines, such as tumor necrosis factor-α (TNF-α), triggering a systemic inflammatory response (Margraf et al., 2020). Non-opioid drugs have demonstrated efficacy in attenuating these inflammatory responses. For instance, the intraoperative administration of dexmedetomidine during thoracoscopic lung cancer surgery has been shown to reduce surgical inflammation, oxidative stress, and postoperative pain, thereby promoting recovery without increasing the risk of adverse events or complications (Zhong et al., 2024). Similarly, Liu et al. reported that esketamine effectively reduces postoperative pain scores, serum IL-6 levels at 24 and 48 h, and the incidence of postoperative delirium in gastrointestinal surgery patients (Liu et al., 2024).

#### 3.1.3 Postoperative gastrointestinal dysfunction

Gastrointestinal surgery patients are particularly susceptible to postoperative complications, including ileus, intestinal obstruction, and a high incidence of PONV. These issues not only compromise patient comfort but also delay oral intake and mobilization, thereby impeding the implementation of ERAS protocols.

OFA circumvents the inhibitory effects of opioids on intestinal motility, facilitating the early recovery of gastrointestinal function. Studies have shown that OFA reduces PONV incidence across various surgical procedures, promotes gastrointestinal recovery (Wang et al., 2024; Feng et al., 2024; Luo et al., 2025), and specifically enhances postoperative gastrointestinal function in gastrointestinal surgeries (Ziemann-Gimmel et al., 2014; Zhou et al., 2024).

### 3.2 Application of OFA in gastrointestinal surgery

The extensive trauma inherent to gastrointestinal surgery and the requirements of ERAS underscore the limitations of traditional opioid-based anesthesia. OFA, by employing multimodal analgesia techniques, not only satisfies analgesic requirements but also mitigates gastrointestinal dysfunction, inflammatory responses, and adverse effects. Consequently, OFA has emerged as an essential optimization strategy for accelerating recovery in gastrointestinal surgery (Table 1).

**TABLE 1:** OFA strategies in gastrointestinal surgery.

Surgery type	Regional nerve block strategies	GA induction	GA Maintenance	Postoperative analgesia
Laparoscopic radical colectomy (An et al., 2022)	Ultrasound-guided bilateral paravertebral block (0.5% ropivacaine plus 0.2 μg/kg Dex)	Dex (0.6 μg/kg, 10 min), Prop (2 mg/kg), Ketorolac (30 mg), Cisatracurium (0.2 mg/kg)	Dex (0.5 μg/kg/h), Sevoflurane (1%–3%), Cisatracurium (2–4 mg per 30 min)	PCA (6 μg·kg^−1^ Dex and 180 mg Ketorolac added to 100 mL of saline at 2 mL/h and the lock time was 15 min)
Bariatric surgery (Ziemann-Gimmel et al., 2014)	Not applied	Dex (0.5 ug/kg, 10 min), Midazolam (2 mg), Prop (1–2.5 mg/kg), Succinylcholine (1–1.5 mg) or Rocuronium (0.5–1 mg/kg)	Dex (0.1–0.3 ug/kg/h), Prop (75–150 mg kg/min), Rocuronium (10–20 mg) or Vecuronium (1–2 mg)	Acetaminophen (1,000 mg) and Ketorolac (30 mg) every 6 h for the first 24 h
Bariatric surgery (Berlier et al., 2022)	Not applied	Clonidin (2 or 3 μg/kg, over 10 min) or Dex (1.4 μg/kg/h, 10 min), Prop (3 mg/kg), Cisatracurium (0.2 mg/kg), Ket (0.5 mg/kg)	Dex (0.5–1 μg/kg/h), Lidocaine (1.5 mg/kg, over 10 min) followed by a continuous infusion of lidocaine 2 mg/kg/h, Volatile anesthetics or Prop	Ketoprofen, Nefopam, and Tramadol were used, Morphine was provided as a rescue analgesic if needed
Bariatric surgery (Perez et al., 2024)	Not applied	Dex (1 μg/kg, over 10 min), Prop (2–3 mg/kg), lidocaine (1.5 mg/kg), Ket (0.5 mg/kg)	Dex (0.3–0.5 μg/kg/h), Sevoflurane, Lidocaine (2 mg/kg/h)	Fentanyl (25–50 μg boluses, 250 μg maximum) and/or hydromorphone (0.5 mg boluses, 2 mg maximum)
Bariatric surgery (Dagher et al., 2025)	Not applied	Prop, Succinylcholine, Lidocaine (1.5 mg/kg), Ket (0.2 mg/kg), Magnesium sulfate (50 mg/kg)	Dex (0.2–0.5 ug/kg/h), Sevoflurane, Lidocaine (1.5 mg/kg/h), Ket (0.15 mg/kg/h), Magnesium sulfate (8 mg/kg/h), Rocuronium	Paracetamol (1 g, every 6 h), and Ketoprofen (50 mg), Morphine sulphate 0.1 mg/kg subcutaneous every 6 h
Sleeve gastrectomy done (Ibrahim et al., 2022)	Ultrasound-guided bilateral oblique subcostal transverse abdominis plane block (0.25% bupivacaine, 40 mL total)	Dex (0.1 μg/kg, 10 min), Prop (2 mg/kg), Ket (0.5 mg/kg), Cisatracurium (0.15 mg/kg)	Dex (0.5 μg/kg/h), Ket (0.5 kg/h), Lidocaine (1 mg/kg/h), Sevoflurane (1.5%–2%)	Paracetamol (1 g, 6 hourly) and Parecoxib (40 mg, 12 hourly)
Sleeve gastrectomy (Mieszczanski et al., 2023)	Local infiltration (0.25% bupivacaine, 40 mL total)	Dex (1ug/kg, 10 min), Prop (2–2.5 mg/kg), Lidocaine (1.5 mg/kg, 10 min), Ket (0.5 mg/kg), Succinylcholine (1–1.5 mg/kg)	Dex (1ug/kg/h, max), Desflurane, Lidocaine (3 mg/kg/h, max), Rocuronium or Cisatracurium	Paracetamol (1 g), Metamizole (1 g, every 6 h), and oxycodone (bolus 2 mg, lockout 10 min)
Sleeve Gastrectomy (Zhou et al., 2024)	Bilateral TAP block (Ropivacaine 0.3%, 20 mL/side), Local anesthesia (Ropivacaine)	Dex (0.5 μg/kg, 10 min), Esk (0.5 mg/kg), Midazolam (0.05 mg/kg), Prop (1–2 mg/kg), Rocuronium (0.6 mg/kg)	Dex (0.2–0.3 μg/kg/h), Esk (0.3 mg/kg/h), Prop (2–3 mg/kg/h), Sevoflurane (0.8%–1%), Cisatracurium (0.04–0.05 mg/kg/h)	VAS value was 7 or above (Tramadol 50 mg), VAS value was 4–7 or in need of analgesia (Flurbiprofen axetil, 50 mg)
Sleeve Gastrectomy (Song et al., 2025)	Ultrasound-Guided TAP Block (0.25% Ropivacaine, 30 mL/side)	Flurbiprofen axetil (50 mg), Dex (1 μg/kg, 10 min), Midazolam (2 mg), Prop (2 mg/kg), Esk (0.5 mg/kg), and Rocuronium (0.6–1 mg/kg)	Prop, Esk and Dex mixture (Esk 50 mg + Dex 150 ug + 0.9% saline into 50 mL) 0.1–0.2 mL/kg/h	PCA (Sufentanil 100 μg + Dex 0.2 mg + Ondansetron 8 mg + 0.9% saline into 100 mL), Flurbiprofen axetil (50 mg, twice daily)

GA, general anesthesia, Dex, Dexmedetomidine, Prop, Propofol, Ket, Ketamine, Esk, Esketamine, VAS, visual analogue scale; TAP, transversus abdominis plane; PCA, patient controlled analgesia.

Unless specified otherwise, the route of administration is intravenous.

However, the successful application of OFA requires the development of individualized anesthetic plans tailored to each patient’s specific clinical circumstances.

### 3.3 Key clinical advantages of OFA in gastrointestinal ERAS

The implementation OFA within gastrointestinal ERAS protocols has demonstrated significant clinical benefits across multiple dimensions, notably enhancing postoperative recovery. This section focuses on four key areas: gastrointestinal function recovery, pain management, reduction of adverse reactions, and patient satisfaction.

#### 3.3.1 Gastrointestinal function recovery

Postoperative gastrointestinal function recovery is often delayed following gastrointestinal surgeries, leading to considerable patient discomfort, prolonged hospital stays, and increased healthcare costs. When integrated with the principles of ERAS, OFA optimizes perioperative management by reducing opioid use. This reduction minimizes the inhibitory effects of opioids on gastrointestinal motility, thereby facilitating quicker recovery of gastrointestinal function.

Studies have demonstrated that intraoperative administration of dexmedetomidine in elderly patients undergoing abdominal surgery significantly shortens the median time to first flatus and bowel movement, as well as the median length of hospital stay (Lu et al., 2021). Similarly, a randomized controlled trial involving patients undergoing laparoscopic colorectal surgery found that continuous intraoperative infusion of esketamine effectively promoted postoperative intestinal function recovery (Sun et al., 2023).

Postoperative pain resulting from noxious stimuli has the potential to increase sympathetic nervous system activity, which may subsequently impede the recovery of gastrointestinal motility. Additionally, the administration of opioid analgesics can further prolong the restoration of normal gastrointestinal function. To address this, selecting appropriate nerve block techniques based on the surgical site such as the transversus abdominis plane block, paravertebral block, erector spinae plane block, Stellate Ganglion Block, and femoral nerve block can provide effective postoperative analgesia while reducing opioid consumption, promote the recovery of gastrointestinal function. For instance, in laparoscopic gynecological surgeries, patients receiving OFA demonstrated significantly improved postoperative analgesia, a lower incidence and severity of postoperative nausea and vomiting (PONV), and faster time to first flatus compared to those receiving opioid-based anesthesia (Chen et al., 2022).

These findings underscore that perioperative pain management strategies aligned with ERAS principles-incorporating opioid-free analgesic regimens and multimodal analgesia techniques-can effectively reduce opioid use and its associated adverse effects, thereby accelerating the recovery of gastrointestinal function.

#### 3.3.2 Pain management

Effective pain management after gastrointestinal surgery is crucial. Adequate postoperative analgesia not only alleviates patient discomfort but also reduces the risk of pain-related complications, such as restricted breathing, pulmonary issues, and deep vein thrombosis. It further facilitates early mobilization, recovery of gastrointestinal function, and accelerates overall recovery. In gastrointestinal enhanced recovery surgery, OFA achieves effective postoperative pain control through the use of various non-opioid drugs and regional block techniques.

For instance, in total hip replacement surgeries, OFA significantly reduces postoperative opioid consumption, pain scores, and hospital stays compared to opioid-based strategies, while minimizing opioid-related side effects. Reported adverse effects were minimal, with no clinical complications observed, highlighting the efficacy of OFA in postoperative pain management (Urvoy et al., 2021). Similarly, in laparoscopic sleeve gastrectomy, patients in the OFA group reported significantly lower postoperative Visual Analog Scale (VAS) pain scores compared to the control group. Additionally, the proportion of patients requiring rescue analgesia was significantly lower (Dagher et al., 2025). These findings further emphasize the advantages of OFA in managing postoperative pain.

#### 3.3.3 Incidence of adverse reactions

While opioids are effective for postoperative pain management, they are associated with adverse effects such as nausea, vomiting, constipation, respiratory depression, itching, and urinary retention. These side effects can prolong hospital stays and increase healthcare costs. By eliminating opioid use, OFA significantly reduces the incidence of these adverse reactions while maintaining effective analgesia.

For example, studies on bariatric and thoracoscopic surgeries have demonstrated that the incidence of PONV is significantly lower in the OFA group compared to the opioid-based anesthesia group (Feng et al., 2024; Ziemann-Gimmel et al., 2014). Furthermore, multiple studies have shown that OFA improves postoperative recovery quality and accelerates recovery across various surgeries, including breast surgery (Zhang Q. et al., 2023), cholecystectomy (Hao et al., 2023), sinus surgery (Zhou et al., 2023), and kidney surgery (Gao et al., 2024).

These findings underscore the ability of OFA to reduce opioid-related adverse reactions while improving the overall quality of postoperative recovery.

#### 3.3.4 Patient satisfaction

Patient satisfaction is a critical indicator of anesthesia effectiveness. The benefits of OFA in promoting gastrointestinal function recovery, alleviating pain, and reducing adverse reactions enhance patient comfort and the overall recovery experience, ultimately improving satisfaction levels.

In studies on bariatric surgery, patients who received OFA reported higher satisfaction scores, with significantly more patients rating their satisfaction as high (Dagher et al., 2025). These findings suggest that OFA better addresses patient needs, delivering higher-quality medical care and contributing to an improved recovery experience.

## 4 Challenges and limitations of OFA

While OFA has demonstrated numerous advantages in gastrointestinal enhanced recovery surgery, its practical application is hindered by several challenges and limitations, which significantly constrain its broader adoption and clinical implementation.

### 4.1 Uncertainty in drug selection and combination

The challenges in selecting and combining drugs for OFA primarily stem from the diverse mechanisms of action of non-opioid drugs, the complexity of clinical scenarios, and the absence of standardized protocols. These challenges can be explored from the following perspectives:

First, a significant challenge lies in the limited efficacy of single drugs and the unpredictable synergistic effects of combination therapies. Non-opioid drugs achieve analgesic, sedative, or nociceptive-inhibitory effects through distinct molecular targets; however, a single drug is often insufficient to meet the multifaceted demands of the entire anesthesia process. Moreover, drug combinations exhibit varying degrees of synergy, which can also heighten the risk of adverse effects. For instance, in gynecological laparoscopic surgery, opioid-free anesthesia has been shown to produce comparable outcomes to traditional opioid-based anesthesia in terms of postoperative nausea and vomiting, postoperative pain, and morphine consumption. However, it has also been associated with prolonged postoperative sedation and extended recovery room stays. This inherent unpredictability in balancing “complementary benefits” with “cumulative adverse effects” complicates clinical decision-making regarding optimal dosing ratios and administration timing in combination therapy.

Second, individual variability in patient responses to drugs further complicates the selection process. Factors such as age, weight, underlying conditions (e.g., hypertension, diabetes), and preoperative pain status significantly influence the pharmacokinetics and pharmacodynamics of non-opioid drugs. For example, in elderly patients and those with hypoalbuminemia, the elimination half-life and context-sensitive half-life of dexmedetomidine are prolonged (Iirola et al., 2012). Additionally, studies have demonstrated that the ED95 of dexmedetomidine for inducing mild sedation is 0.38 μg/kg in patients over 65 years old, compared to 0.57 μg/kg in patients aged 45–64 (Kim et al., 2015). These findings highlight the necessity of individualized adjustments to OFA regimens to accommodate patient-specific characteristics.

Finally, the lack of standardized guidelines or protocols for clinical practice management further exacerbates the challenges associated with OFA. This absence of uniformity limits the guidance available to anesthesiologists, increases uncertainty, and heightens risks during OFA implementation.

### 4.2 Insufficient evidence of effectiveness

Although some studies have emphasized the potential advantages of OFA, the evidence supporting its benefits remains insufficient. Many studies are constrained by small sample sizes, suboptimal study designs, and the lack of large-scale, multicenter, high-quality clinical trials to robustly validate its efficacy and safety. Furthermore, heterogeneity in study outcomes complicates the overall assessment of OFA’s effectiveness.

For instance, studies report significant benefits of OFA in pain control and the recovery of gastrointestinal function (Lu et al., 2021; Sun et al., 2023; Chen et al., 2022). However, other studies have shown that OFA, when compared to traditional opioid-based anesthesia, does not improve anesthesia quality in patients (Chassery et al., 2024; Perez et al., 2024). Moreover, it may contribute to additional adverse effects, such as bradycardia, hemodynamic instability, and prolonged the recovery room stays (Beloeil et al., 2021; Mieszczanski et al., 2023; Zhang et al., 2025). This inconsistency underscores the need for rigorous research to elucidate the specific benefits, limitations, and appropriate applications of OFA in gastrointestinal enhanced recovery surgery.

### 4.3 Potential adverse reactions

Although OFA seeks to minimize opioid-related adverse effects, the use of multiple adjuvant analgesic drugs introduces the risk of potential adverse reactions. For example, dexmedetomidine is associated with bradycardia, hypotension, and excessive sedation (Beloeil et al., 2021; Feng et al., 2024; Mieszczanski et al., 2023), necessitating vigilant monitoring of patients’ vital signs and timely dose adjustments. Similarly, lidocaine, particularly when administered in high doses or over extended durations, may result in local anesthetic systemic toxicity, manifesting as central nervous system excitation or depression. Additionally, esketamine has been linked to dissociative symptoms and hallucinations, which may lead to psychiatric adverse effects.

These potential adverse reactions significantly increase the complexity of clinical management, underscoring the need for clinicians to possess substantial experience and expertise to promptly recognize and effectively address such issues.

### 4.4 Challenges in clinical implementation

The clinical implementation of OFA presents several challenges. Firstly, OFA demands advanced expertise from healthcare providers, including a thorough understanding of the pharmacological properties, administration techniques, and potential adverse effects of non-opioid drugs, as well as proficiency in regional block techniques. This necessitates systematic training and education; however, in primary healthcare institutions or resource-limited regions, constrained resources and a lack of expertise hinder the ability to meet these requirements.

Secondly, OFA often involves the use of equipment, which contributes to increased healthcare costs and resource utilization. During the implementation of OFA, electroencephalography monitoring often reveals depth of anaesthesia values exceeding expected levels (Mogianos and Persson, 2025). To ensure patient safety, supplementary monitoring techniques, such as pain assessment devices, become necessary (An et al., 2017; Meijer et al., 2020). However, these interventions inevitably result in increased healthcare costs and resource utilization. Furthermore, patient awareness and acceptance play a crucial role in its clinical implementation. Some patients may harbor doubts or concerns regarding this novel anesthetic approach, further complicating its adoption in practice.

Addressing these obstacles is critical to improving the clinical adoption rate of OFA and unlocking its full potential to enhance perioperative care.

## 5 Prospects and future directions for OFA in gastrointestinal ERAS

Although OFA presents several challenges in its application to gastrointestinal enhanced recovery surgery, its distinct advantages and potential underscore a promising future. The following strategies may further facilitate the advancement of OFA in this field.

### 5.1 Optimizing drug combinations and protocols

To address the existing uncertainty regarding drug selection and combinations, future research should prioritize the optimization of OFA protocols. Comprehensive clinical trials and experimental studies are essential to investigate the synergistic effects and optimal compatibility of various drugs, with the goal of identifying the most effective combinations and dosages tailored to specific surgical procedures and individual patient profiles. Personalized OFA protocols should account for factors such as patient age, comorbidities, and physical status to maximize anesthetic efficacy and safety. Furthermore, advancements in pharmaceutical research may facilitate the development of novel non-opioid drugs, thereby broadening the therapeutic arsenal available for OFA.

### 5.2 Conducting large-scale, multicenter studies

To address the current lack of robust evidence, large-scale, multicenter clinical studies are crucial. Larger sample sizes increase the reliability and generalizability of findings, while multicenter designs enhance representativeness and minimize research bias. Such studies would enable a more accurate assessment of the efficacy and safety of OFA in gastrointestinal enhanced recovery surgery, thereby clarifying its clinical value and applicability. Additionally, long-term follow-up studies are needed to evaluate the sustained effects of OFA, offering valuable insights to guide clinical practice.

### 5.3 Enhancing training for healthcare providers

The successful clinical implementation of OFA relies heavily on the expertise of healthcare providers, making enhanced training an essential priority. Systematic theoretical training should ensure a comprehensive understanding of OFA, including the pharmacological properties, mechanisms of action, administration techniques, and potential adverse effects of non-opioid drugs. Practical training should emphasize proficiency in techniques such as regional anesthesia and nerve blocks. Regular training programs, workshops, and academic exchanges can further support healthcare providers in continuously refining their skills, thereby ensuring the safe and effective application of OFA in clinical practice.

### 5.4 Combining OFA with other treatment modalities

Future strategies should focus on integrating OFA with other therapeutic approaches to further improve patient recovery and quality of life. For instance, combining OFA with rehabilitation therapy could facilitate targeted recovery training during the early postoperative period, promoting the restoration of physical function. Additionally, integrating psychological interventions may help alleviate patient anxiety and fear, enhancing psychological resilience. By incorporating multiple treatment modalities, synergistic effects can be achieved, providing more comprehensive and high-quality medical care while advancing the development of gastrointestinal enhanced recovery surgery.

## 6 Conclusion

OFA has demonstrated significant advantages in gastrointestinal enhanced recovery surgery by promoting gastrointestinal function recovery, enhancing pain management, reducing the incidence of adverse reactions, and improving patient satisfaction. Evidence indicates that OFA shortens the time to first flatus and bowel movement, reduces hospital stays, effectively controls postoperative pain, and minimizes opioid consumption along with its associated adverse effects. Furthermore, OFA has been shown to decrease complications such as PONV and respiratory depression, thereby enhancing patient recovery experiences and satisfaction.

This review provides a comprehensive analysis of the application of OFA in gastrointestinal enhanced recovery surgery, offering valuable insights for clinicians. By summarizing relevant studies, it clarifies the advantages and challenges of OFA, supporting the development of tailored anesthetic approaches to meet individual patient needs and improve the overall quality of care. Additionally, this review outlines key directions for future research, fostering the ongoing development and refinement of OFA techniques.

Despite the numerous benefits associated with OFA, its clinical application warrants careful consideration. The current uncertainties regarding drug selection and the limited availability of robust evidence highlight the need for meticulous patient evaluation and the formulation of personalized anesthetic protocols. Moreover, enhanced training for healthcare providers is essential to ensure the safe and effective implementation of OFA in clinical practice.

Future large-scale, multicenter studies are crucial to establish optimal protocols and indications for OFA, facilitating its broader adoption in gastrointestinal enhanced recovery surgery. Overall, OFA presents promising opportunities in this field. With further research and advancements in technology, OFA is poised to play an increasingly pivotal role, ultimately providing patients with safer, higher-quality medical care.
